# Short Communication: Prevalence of *Listeria monocytogenes* in Raw Milk of Healthy Sheep and Goats

**DOI:** 10.1155/2022/3206172

**Published:** 2022-05-19

**Authors:** Fatemeh Sepahvand, Ehsan Rashidian, Amin Jaydari, Heidar Rahimi

**Affiliations:** Department of Pathobiology, Faculty of Veterinary Medicine, Lorestan University, Khorramabad, Lorestan, Iran

## Abstract

*Listeria monocytogenes*, one of the most important bacterial pathogens transmitted through milk, causes listeriosis in humans and animals. This study aimed to investigate the prevalence of *L. monocytogenes* in raw milk of healthy sheep and goats in the west of Iran (Lorestan Province) by touchdown PCR (TD-PCR). *Listeria* spp. were found in milk samples taken from 21 sheep (29.16%) and 3 goats (10.71%) whereas *L. monocytogenes* was isolated from milk samples taken from 4 sheep (5.55%) and 1 goat (3.75%). The results showed that there was a significant difference between sheep and goats in the prevalence of *Listeria* spp. in their raw milk (*p* < 0.05), but no significant difference was observed between them in the prevalence of *L. monocytogenes*. The study findings suggested that the raw milk of healthy sheep and goats was infected with *L. monocytogenes* and warned of the risk of human infection with listeriosis following consumption of raw and unpasteurized milk.

## 1. Introduction

Listeriosis is one of the most important bacterial infections worldwide. According to the reports published by the Centers for Disease Control and Prevention (CDC), it is estimated that listeriosis is annually the cause of 1,600 cases of disease and 260 deaths [1]. As a severe food-borne illness, listeriosis is often caused by eating foods contaminated with *L. monocytogenes*. Raw milk is one of the most common environments for the transmission of *L. monocytogenes* [2].

Sheep milk was formerly known as a possible cause of listeriosis [3]. Since milk and dairy products are of high nutritional value, they are suitable environments for the proliferation and growth of microorganisms including pathogenic bacteria. *L. monocytogenes* is usually found in dairy products, on farms, and in dairy processing plants [2]. There are reports on the intermittent, asymptomatic shedding of *L. monocytogenes* in the milk of goats and sheep [4, 5]. It is hence necessary to identify infected animals in order to prevent the possible prevalence of listeriosis [3].

Currently, the standard method for diagnosing *L. monocytogenes* is culture, but because antibiotics are used in animal feed and this bacterium is intracellular, the sensitivity of the culture is greatly reduced. In addition, isolation and identification of *L. monocytogenes* using culture media and biochemical methods is a time-consuming process. Therefore, using the PCR technique to identify this bacterium in a clinical sample is a valuable method. Listeriolysin O (LLO) is a secretory protein encoded by the *hlyA* gene and one of the main factors responsible for the virulence of *L. monocytogenes* so that its isolation from food products can indicate the presence of *L. monocytogenes* [6, 7]. Most studies have investigated the prevalence of *L. monocytogenes* in cow's milk and a few have dealt with the prevalence of this bacterium in sheep and goat raw milk. Lorestan with a livestock population of about 3750,000 heads, is one of the top 5 provinces in Iran in the field of animal husbandry. This study hence aimed to employ the TD-PCR method for the direct detection of *L. monocytogenes* to determine its prevalence in sheep and goat raw milk in Lorestan Province, Iran.

## 2. Materials and Methods

### 2.1. Milk Samples

From January to February 2020 (winter season), 100 milk samples were randomly collected from 72 sheep and 28 goats in livestock farms in four regions, namely, Aleshtar and Azna (cold regions), and Khorramabad and Kuhdasht (tropical regions) of Lorestan province (Iran). 50 milliliters of each sample was gathered in tubes under aseptic conditions and transferred to the laboratory on an icebox. The samples were stored at −20°C for molecular experiments until processing.

### 2.2. DNA Extraction

Frozen milk was thawed at room temperature, and 10 mL of each sample was added to a 10 mL tube. The tubes were centrifuged at 13,000 × *g* for 15 min. Then, with a sterile swab, the fatty top layer was removed and the liquid around was poured. Next, 200 *μ*L of precipitate were transferred to a separate tube each to extract DNA using a Blood Genomic DNA Extraction Mini Kit (Favorgen, Taiwan) according to the manufacturer's instructions with a few modifications. The quality and amount of extracted DNA was measured by using a Nanodrop spectrophotometer (Thermo Scientific, Waltham, USA). Finally, the extracted DNA was stored at −20°C for the later use in PCR.

### 2.3. DNA Amplification and Detection of Polymerase Chain Reaction (PCR) Products

The isolates were identified using the TD-PCR method for the identification of the *16S rRNA* gene (553 bp) with a pair of primers, forward (5′-CCT TTG ACC ACT CTG GAG ACA GAG C-3′) and reverse (5′-AAG GAG GTG ATC CAA CCG CAC CTT C-3′) designed by Lantz et al. [8] and *hlyA* gene (702 bp) with a pair of primers, forward (5′-CCTAAGACGCCAATCGAA-3′) and reverse (5′-AAGCGCTTGCAACTGCTC-3′) designed by Border et al. [9] amplifiers specifically for *Listeria* spp. and *L. monocytogenes*, respectively. The primer sequences were provided by Takapou Zist Company, Tehran, Iran.

Touchdown (TD)-PCR amplification was performed by using a PCR master kit (Ampliqon Taq DNA Polymerase Master Mix RED 1.25 mL, Ampliqon Denmark) with 25 *μ*L mixtures containing 12.5 *μ*L of 2X master mix, 0.5 *μ*L of each primer, and 4 *μ*L of the extracted DNA. In this study, DNA obtained from *L. monocytogenes* (laboratory control strain: (PTCC 1294) Iran Scientific and Industrial Research Center) and sterile water were used as the positive and negative controls, respectively. Furthermore, the amplification was conducted by using a Bio-Rad thermocycler (Model T-100, USA) under the following conditions: A for *Listeria* spp. and B for *L. monocytogenes*.

- The initial step of 94°C for 5 min, followed by 5 cycles of 94°C for 1 min, annealing temperatures starting at 64°C for 45 seconds (decreasing 1°C/cycle), and finally, at 72°C for 30 seconds for the extension. This step was followed by 30 cycles of 94°C for 30 seconds, 60°C for 45 seconds, 72°C for 45 seconds, and finally, 72°C for 10 min.
- The initial step of 95°C for 4 min, followed by 5 cycles of 95°C for 1 min, annealing temperatures starting at 55°C for 45 seconds (decreasing 1°C/cycle), and finally, at 72°C for 1 min for the extension. This step was followed by 30 cycles of 95°C for 1 min, 52°C for 45 seconds, 72°C for 2 min, and finally, 72°C for 8 min.

The PCR products were separated in a 1.2% (w/v) agarose gel (Merck, Germany) containing 2.5 *μ*g/mL gel stain (Smobio, Taiwan). Electrophoresis was performed in 0.5x Tris/Borate/EDTA (TBE) buffer for one hour at 100 V. The resulting PCR products were visualized under a UV transilluminator (E-Box, Iran) and the 100 bp DNA ladder (Smobio, Taiwan) plus was used as the molecular size marker.

### 2.4. Statistical Analysis

The obtained data were analyzed by IBM SPSS 20 for Windows (SPSS Inc., Chicago, IL, USA). *p* value < 0.05 was considered significant.

## 3. Results

**Figure 1:**
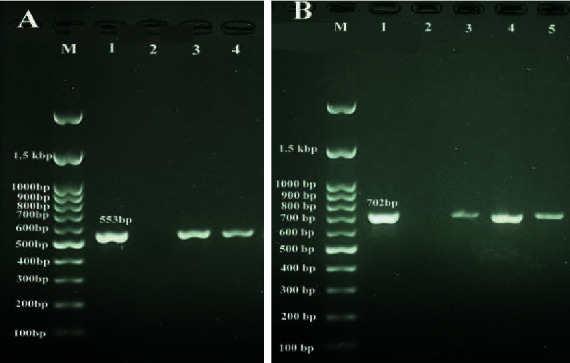
Touchdown PCR assay for the detection of *Listeria* spp. (a) and *L. monocytogenes* (b) in raw milk samples. A: Lane M: standard DNA marker; Lane 1: positive control; Lane 2: negative control; Lanes 3 and 4: positive samples for *Listeria* spp. B: Lane M: standard DNA marker; Lane 1: positive control; Lane 2: negative control; Lanes 3–5: positive samples for *L. monocytogenes*.

In this study, 100 samples of raw milk were taken from 72 sheep and 28 goats to detect *L. monocytogenes*. *Listeria* spp. identified using *16s rRNA* gene sequencing was found in milk samples taken from 21 sheep (29.16%) and 3 goats (10.71%), and *L. monocytogenes* identified by detecting the *hlyA* gene was isolated in milk samples taken from 4 sheep (5.55%) and 1 goat (3.75%) (Figure 1). Among the regions, Kuhdasht (tropical region) livestock were more infected with *Listeria* spp. than other cities and Azna (cold region) livestock were more infected with *L. monocytogenes* (Table 1). There was a significant difference observed between milk samples taken from sheep and goat in the prevalence of *Listeria* spp. (*p* < 0.05).

## 4. Discussion

Listeriosis is an infectious disease in humans and animals in which 99% of cases are caused by consumption of foods contaminated with *L. monocytogenes* and is rarely transmitted from the environment [10]. Raw milk is one of the most common substrates for the transmission of *L. monocytogenes* as studies have shown that *Listeria* spp. isolates can be usually found in raw milk specimens [10–12]. Lorestan province has cold and tropical regions due to its geographical location. Also, in this province, more than 80% of the livestock population is sheep in order to preserve pastures and oak trees. Therefore, in the present study, milk samples were collected from both tropical and cold regions and were more than sheep. Various studies indicated that the TD-PCR method increases both the specificity and sensitivity of the PCR protocol, since the content of unwanted in the DNA extracted from the milk is high. Therefore, using TD-PCR is considered the suitable method for increasing the template copy number leading to an increase in the yield of amplicons [13–16]. The results of the present study using TD-PCR showed that the prevalence of the *Listeria* spp. isolates was equal to 29.16% and 10.71% in samples isolated from sheep and goat milk, respectively. In addition, 5 isolates (20.83%) of *Listeria* spp. belonged to *L. monocytogenes*. The prevalence of this bacterium in raw milk samples of sheep and goat was 5.55% and 3.57%, respectively. Many studies have been conducted around the world on the prevalence of *L. monocytogenes* in cow's milk. Their findings indicated that the prevalence of this bacterium was 4% in the Netherlands [17], 4% in the USA [18], 2% in Turkey [19], 1.1% in Brazil [20], 1.7% in Italy [21], 3.6% in Ethiopia [22], 11% in Iraq [23], 1.7% in Iran [24], and 1.1% in Iran [12]. Nevertheless, few studies have investigated the prevalence of *L. monocytogenes* in sheep and goat raw milk.

Studies conducted by Durmaz et al. [19] in Turkey showed that the prevalence rates of *L. monocytogenes* in sheep and goat raw milk were 2.7% and 0%, respectively. In another study conducted by Abbas and Jaber [23] in Iraq, the prevalence of *L. monocytogenes* in sheep milk was 8%. The study conducted by Rahimi et al. [12] in Isfahan Province, Iran, demonstrated that the prevalence rates of *Listeria* spp. in sheep and goat raw milk were 22.6% and 6.7%, respectively, and the prevalence rates of *L. monocytogenes* were 6.5% and 1.7%, respectively. In another study conducted by Jamali et al. [25] in Alborz Province, Iran, the prevalence rates of *Listeria* spp. in sheep and goat raw milk were 16.4% and 4.9%, respectively, and the prevalence of *L. monocytogenes* was 4% in sheep and goat raw milk. In another study by Osman et al. [26] in Egypt, the prevalence rates of *Listeria* spp. in sheep and goat raw milk were 3.9% and 5.6%, respectively, and the prevalence rates of *L. monocytogenes* were 1% and 1.9%, respectively.

Consistent with the findings of Durmaz et al. [19], Rahimi et al. [12], and Jamali et al. [25], this study showed that the prevalence of *Listeria* spp. was higher in sheep milk than in goat milk and there was a significant difference between milk specimens taken from sheep and goats in this regard. By contrast, Osman et al. [26] in Egypt reported that the prevalence of *Listeria* spp. and *L. monocytogenes* was higher in goat milk than in sheep milk. This study, however, consistent with the results of Rahimi et al. [12] and Jamali et al. [25], demonstrated that the prevalence of *Listeria* spp. and *L. monocytogenes* was higher in sheep milk than in goat milk in Iran. This can be attributed to the higher health status of the udder in goats than in sheep as well as the higher tendency of sheep to graze and rest in compact groups.

Lorestan province with a high livestock population has tropical and cold regions throughout the year. As, the two regions of Aleshtar and Azna are among the cold regions, while Khorramabad and Kuhdasht are two tropical regions of the province. The present study showed that the samples isolated from Kuhdasht as a tropical region are more contaminated with *Listeria* spp. than other regions of the province. However, samples isolated from Azna as a cold region are more contaminated with *L. monocytogenes* than in other regions. Considering that all milk samples were collected in winter, but there is no significant difference in the prevalence of *Listeria* spp. and *L. monocytogenes* between tropical and cold regions of the province.

There are various reports that the feeding of livestock on poor-quality silage and poor milking hygiene are two important sources of milk contamination with *Listeria* spp. especially *L. monocytogenes* [27, 28]. The selected livestock in this study had no history of feeding on silage, and also milking cartridges were disinfected with 70% alcohol. Therefore, the isolates identified in this study were not caused by poor hygiene, and the animals were already asymptomatic carriers of listeriosis.

The study findings are also consistent with those of Durmaz et al. [19], Rahimi et al. [12], Mahmoodi [24], and Jamali et al. [25]. Although most studies have focused on the prevalence of *Listeria* spp. in cow's milk, the prevalence of this bacterium is higher in sheep and goat raw milk. This indicates the importance of pasteurizing sheep and goat milk. Various studies have indicated that this bacterium can survive at pasteurization and even freezing temperatures for a long time depending on its strain type and concentration in milk [18, 29]. The presence of bacteria is a serious risk of infection from eating contaminated food even at low prevalence and concentration of the pathogen, especially when unpasteurized dairy products are consumed [26].

Since there are reports of asymptomatic carriage of *L. monocytogenes* [7, 26] and sheep and goat milk is used for preparing local cheese in most rural areas of Iran, the implementation of systematic monitoring in dairy herds is relevant to identify infected animals and prevent and control the presence of *L. monocytogenes*. Other studies should also be conducted to determine circulating variants and identify resistant strains, and even the inclusion of innovative alternatives for the treatment of milk from positive animals such as the use of lactose oxidase which was recently reported by Flynnet et al. [30] should be performed. All of the above prevent outbreaks among the population of consumers of milk or its by-products, when they do not go through the pasteurization process.

## Figures and Tables

**Table 1:** Distribution and prevalence of *Listeria* spp. and *L. monocytogenes* in milk samples.

Region	Animal	Number	*Listeria* spp.		*L. monocytogenes*	
			Positive	Percentage	Positive	Percentage
Azna	Sheep	15	4	26.66	1	6.66
	Goat	10	1	10	1	10

Aleshtar	Sheep	16	4	25	1	6.25
	Goat	7	1	14.28	0	0

Khorramabad	Sheep	23	6	26.08	1	4.34
	Goat	4	0	0	0	0

Kuhdasht	Sheep	18	7	38.88	1	5.55
	Goat	7	1	14.28	0	0

Total	Sheep	72	21	29.16	4	5.55
	Goat	28	3	10.71	1	3.57

## Data Availability

No additional data were used to support this study.
